# Association of preoperative muscle-adipose index measured by computed tomography with survival in patients with esophageal squamous cell carcinoma

**DOI:** 10.1186/s12957-024-03338-0

**Published:** 2024-02-22

**Authors:** Danqi Qian, Peipei Shen, Jiahao Zhu, Shengjun Ji, Yan Kong

**Affiliations:** 1 Department of Radiotherapy and Oncology, The Affiliated Hospital of Jiangnan University, Wuxi, Jiangsu 214000 People’s Republic of China; 2 Department of Radiotherapy and Oncology, The Affiliated Suzhou Hospital of Nanjing Medical University, Gusu School, Nanjing Medical University, Suzhou, Jiangsu 215000 People’s Republic of China

**Keywords:** Muscle-adipose index, Esophageal squamous cell carcinoma, Nutrition indicators, Body composition indicators, Prognosis

## Abstract

**Background:**

Conventional nutritional metrics are closely associated with the prognosis of patients with radically resected esophageal squamous cell carcinoma (ESCC). Nevertheless, the prognostic implications of muscle and adipose tissue composite indexes in ESCC remain unknown.

**Methods:**

We retrospectively analyzed clinicopathological data of 304 patients who underwent resected ESCC. To obtain measurements of the muscle and adipose indexes, preoperative computed tomography (CT) images were used to quantify skeletal-muscle adipose tissue. The diagnostic threshold for muscle-adipose imbalance was determined using X-tile software and used to analyze the association between the muscle-adipose index (MAI) and survival. Instantaneous risk of recurrence was assessed using a hazard function. We constructed a nomogram based on the MAI and other clinical characteristics and established a novel predictive model with independent prognostic factors. The prognostic capabilities of these nomograms were evaluated using calibration curves, receiver operating characteristic (ROC) curves, and decision-curve analysis (DCA).

**Results:**

The overall survival (OS) and disease-free survival (DFS) rates in the muscle-adipose-balanced group were significantly better than those in the muscle-adipose-imbalanced group. Multivariate analyses revealed that the MAI, prognostic nutritional index (PNI), tumor stage, and tumor differentiation were independent prognostic factors for OS and DFS in patients with resected ESCC (*P* < 0.05). The nuclear density curve indicated a lower risk of recurrence for patients in the muscle-adipose-balanced group than that for their imbalanced counterparts. Conversely, the nuclear density curve for PNI was confounded. Postoperative radiotherapy- (RT) benefit analysis demonstrated that patients with ESCC in the muscle-adipose-balanced group could benefit from adjuvant RT.

**Conclusion:**

This study confirmed that preoperative MAI could serve as a useful independent prognostic factor in patients with resected ESCC. A nomogram based on the MAI and other clinical characteristics could provide individualized survival prediction for patients receiving radical resection. Timely and appropriate nutritional supplements may improve treatment efficacy.

**Supplementary Information:**

The online version contains supplementary material available at 10.1186/s12957-024-03338-0.

## Background

Esophageal squamous cell carcinoma (ESCC) is a primary histological subtype of esophageal cancer that accounts for approximately 70–80% of cancer cases in China [1, 2]. Despite advancements in diagnostic and therapeutic strategies, the overall 5-year survival rate of ESCC has only marginally improved, remaining between 15 and 20% [3]. Patients with esophageal cancer are predisposed to nutritional risks owing to metabolic and feeding-related issues. Consequently, the preoperative nutritional status of patients with esophageal carcinoma (EC) is gaining increasing attention, as it has been confirmed to influence prognosis [4]. Therefore, assessing the nutritional status of patients with EC is crucial for risk stratification and provides valuable insights for predicting survival outcomes.

Recent studies have identified a range of nutrition-related markers such as body mass index (BMI), prognostic nutritional index (PNI), and controlling nutritional status (CONUT) score, which are important markers for evaluating the nutritional status of patients with EC prior to surgery [4, 5]. While BMI calculated from measured height and weight do not reflect body composition indicators, two constituents of weight, muscle and adipose tissue, have unique associations with survival outcomes [6]. Additionally, preoperative hematological indicators serve as the basis for serum albumin concentration (ALB), CONUT score, and PNI. A retrospective study demonstrated that EC patients with PNI < 50 or CONUT score ≥ 4 had worse survival [4]. However, these indicators vary and may not comprehensively reflect nutritional fluctuations [7]. Recently, software developments have made it feasible to exploit clinically obtained computed tomography (CT) images to assess body composition. This approach proposes the application of body composition indexes (BCIs), which include the quantity and quality of muscle, and the distribution of adipose tissues, as new indicators to evaluate nutritional status in many malignancies [8]. Sarcopenia and high visceral adipose tissue levels increase cancer-related mortality [4].

This study comprehensively analyzed the relationship between the survival prognosis of patients with resected ESCC and BCI, which combines the muscle and adipose tissues. We compared the prognostic significance of the muscle-adipose index (MAI) and other conventional nutritional indicators in patients with ESCC. Moreover, a predictive nomogram based on the MAI and other clinical characteristics was constructed.

## Methods

### Patients

We recruited 304 patients with ESCC who underwent radical esophagectomy between June 2015 and March 2019 at the Affiliated Hospital of Jiangnan University (187 cases), and the Affiliated Suzhou Hospital of Nanjing Medical University (117 cases). The following case selection criteria were defined: (1) postoperative pathology-confirmed ESCC; (2) R0 excision; (3) radical resection with stages TNM I–III; (4) no distant organ metastasis; and (5) clinicopathological data, including biochemical indicators examined in blood recorded within 3 days prior to surgery, and body composition parameters measured by CT within a month before surgery. Postoperative tumor staging was performed using the TNM staging system (AJCC on Cancer, 8th edition). This study adhered to the Declaration of Helsinki and was approved by the Affiliated Hospital of Jiangnan University and the Affiliated Suzhou Hospital of Nanjing Medical University.

### Nutritional assessment

The traditional nutritional indexes used in this study were defined and calculated as follows: (1) PNI was calculated using the formula PNI = 10 × serum albumin (g/dl) + 0.005 × total lymphocyte count. A cutoff point of 46 was selected [9, 10]. (2) The CONUT score was calculated from the serum albumin, total lymphocyte count, and total cholesterol (TC) levels. Patients were classified into two categories: low CONUT scores (0–4) and high CONUT scores (> 4) [11]. (3) The Naples prognostic score (NPS) was calculated using four serum indicators: ALB, TC, lymphocyte-to-monocyte ratio, and neutrophil-to-lymphocyte ratio. Based on their final scores (0–2), patients were classified into three groups [12]. (4) BMI was calculated as weight/height^2^ (kg/m^2^). (5) Hypoproteinemia was defined as an ALB level of < 35 g/L.

### Body composition assessment

**Fig. 1:**
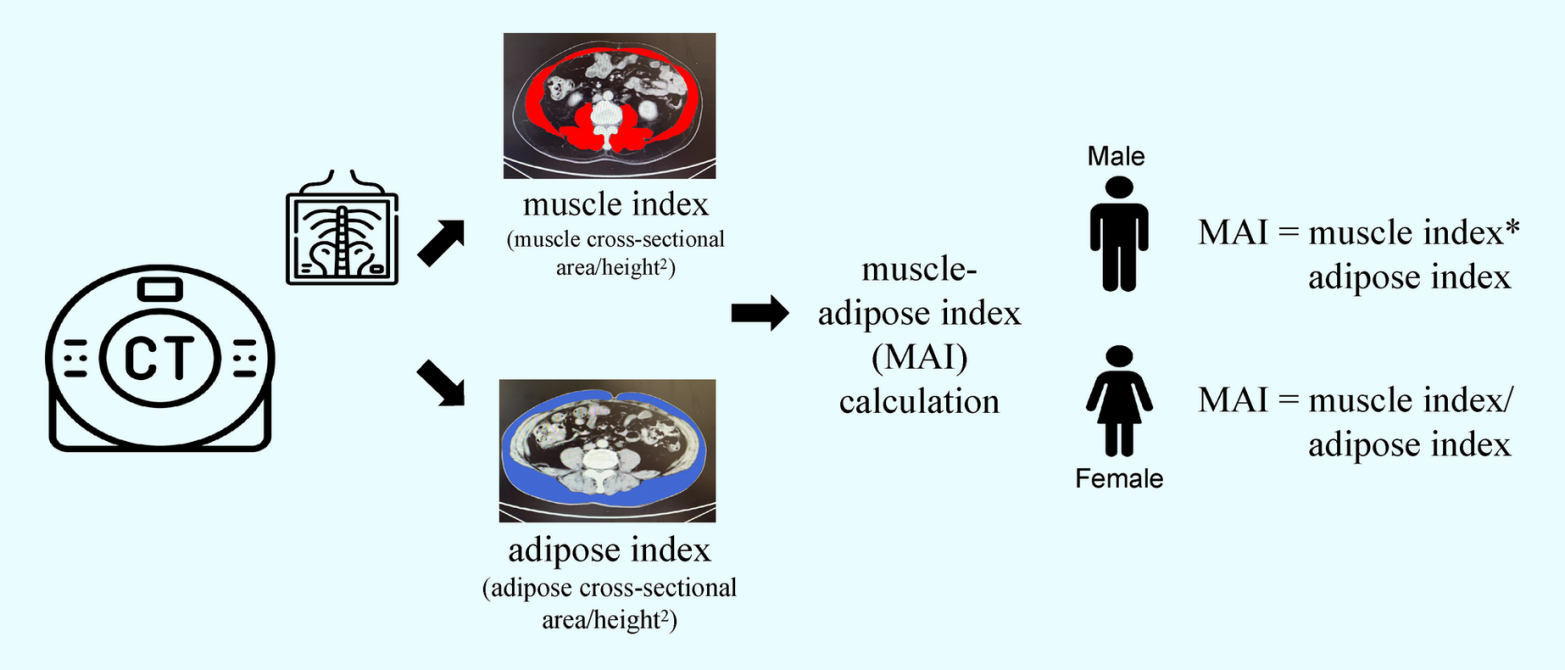
Muscle-adipose index Obtaining and Calculating

The abdominal muscles and adipose tissues were measured using CT scans at the L3 vertebral and umbilical levels, respectively [13, 14]. These muscles include the rectus abdominis, psoas, quadratus lumborum, paraspinal, transverse abdominal, external oblique, internal oblique, and rectus abdominis [7]. The adipose tissues included subcutaneous adipose tissue. Owing to the quantification inaccuracy of visceral adipose tissue, only subcutaneous fat was analyzed in this study [15]. The cross-sectional areas of the muscle and adipose tissues were quantified in square centimeters by two trained researchers, and component discrimination was conducted using 3D Slicer software with tissue-specific Hounsfield units [16]. The square of height was utilized as the standardization metric for body-composition assessment in this study, allowing for the computation of both muscle and adipose indices (cm^2^/m^2^). Considering the different prognostic implications of the adipose index in male and female patients, two distinct definitions have been used for the MAI. For male patients, the formula involved multiplying the adipose index by the muscle index, whereas for female subjects, the ratio of the muscle index to the adipose index was used [7, 17] (Fig. 1). The threshold value of the MAI in the two gender groups was defined using X-tile software. Patients who scored below and above the defined cutoff value were categorized into the muscle-adipose imbalanced and balanced groups, respectively.

### Treatment

The McKeown and Ivor-Lewis procedures with two- or three-field lymph node dissection are the primary surgical options for patients [18–20]. Patients pathologically diagnosed with T4 tumors and/or positive lymph nodes underwent postoperative radiotherapy (RT) (45–50.4 Gy) with or without chemotherapy (cisplatin and fluoropyrimidine) within six weeks of surgery. Some patients with T3 tumors and risk factors are advised to undergo postoperative treatment. In this study, patients received follow-up assessments every three months during the initial two-year period, followed by biannual evaluations over a subsequent three-year duration, and annual evaluations from the fifth year onward. The primary endpoints of the present investigation included overall survival (OS) and disease-free survival (DFS). OS represents the duration between the operation and death or last visit, whereas DFS represents the interval from the surgery for ESCC to the first occurrence or cancer-related death. The final follow-up was conducted in May 2022.

### Statistical analysis

The R software (version 4.2.1 and Medcalc 17.9 (MedCalc Software bvba, Ostend, Belgium) was used for all statistical analyses. Chi-squared tests or Fisher's exact tests were used to analyze categorical variables, whereas continuous variables were evaluated using a t-test. OS and DFS rates were calculated using the Kaplan–Meier method and differences were assessed using log-rank tests. A Cox proportional hazards regression model was used to analyze the relationship between survival and prognostic factors in both univariate and multivariate analyses. The DFS hazard function was used to estimate the recurrence hazard and peak recurrence time, with the event criterion being any recurrence. Hazard rate (HR) was estimated using the kernel smoothing method. Statistical significance was defined as a two-sided *P*-value < 0.05.

## Results

### Patient characteristics

The baseline characteristics of the enrolled patients are shown in Table 1. This study included 304 patients diagnosed with ESCC (204 male and 100 female patients). The average age at diagnosis of all the patients included in this study was 60.5 years, ranging from 30 to 85 years. Of all the patients, 72 (23.7%) were in TNM I stage, 127 (41.8%) were in TNM II stage, and 105 (34.5%) were in TNM III stage. The mean adipose and muscle indexes for all patients were 58.22 cm^2^/m^2^ and 41.63 cm^2^/m^2^, respectively. Upon comparing the clinical characteristics, significant differences were noted in the adipose and muscle indexes across the male and female groups (all *P* < 0.05). Conversely, no notable discrepancies were observed in the other clinicopathological data between the two groups (all *P* > 0.05).

**Table 1:** Baseline clinicopathological characteristics of esophageal squamous cell carcinoma patients

Characteristics	Gender			Muscle-Adipose Index		
	Female (*N* = 100)	Male (*N* = 204)	*P*	Unbalance (*N* = 173)	Balance (*N* = 131)	*P*
Age (years)			0.153			0.623
< 65	56 (56%)	133 (65.2%)		105 (60.7%)	84 (64.1%)	
≥ 65	44 (44%)	71 (34.8%)		68 (39.3%)	47 (35.9%)	
BMI			0.206			0.669
Mean ± SD	23.477 ± 3.567	22.929 ± 3.534		23.185 ± 3.658	23.009 ± 3.409	
Sum of adipose tissue (cm^2^/m^2^)			< 0.001			0.514
Mean ± SD	63.798 ± 9.822	55.497 ± 6.498		58.497 ± 9.837	57.872 ± 6.834	
Sum of muscle (cm^2^/m^2^)			< 0.001			< 0.001
Mean ± SD	37.972 ± 10.292	43.418 ± 8.168		36.200 ± 6.976	48.794 ± 6.694	
Tumor length (cm)			0.086			0.894
Mean ± SD	4.108 ± 1.974	4.501 ± 1.817		4.360 ± 1.848	4.389 ± 1.920	
Vessel invasion			0.989			0.928
Negative	87 (87%)	177 (86.8%)		151 (87.3%)	113 (86.3%)	
Positive	13 (13%)	27 (13.2%)		22 (12.7%)	18 (13.7%)	
Perineural invasion			0.155			0.301
Negative	86 (86%)	160 (78.4%)		144 (83.2%)	102 (77.9%)	
Positive	14 (14%)	44 (21.6%)		29 (16.8%)	29 (22.1%)	
Differentiation			0.128			0.763
Well	11 (11%)	33 (16.2%)		26 (15%)	18 (13.7%)	
Moderate	64 (64%)	138 (67.6%)		112 (64.7%)	90 (68.7%)	
Poor	25 (25%)	33 (16.2%)		35 (20.2%)	23 (17.6%)	
Tumor location			0.232			0.568
Upper	5 (5%)	4 (1.9%)		17 (9.8%)	12 (9.2%)	
Middle	48 (48%)	112 (54.9%)		95 (54.9%)	65 (49.6%)	
Lower	47 (47%)	88 (43.1%)		61 (35.3%)	54 (41.2%)	
TNM stage			0.203			0.665
I	28 (28%)	44 (21.6%)		44 (25.4%)	28 (21.4%)	
II	44 (44%)	83 (40.7%)		72 (41.6%)	55 (42%)	
III	28 (28%)	77 (37.7%)		57 (32.9%)	48 (36.6%)	
Adjuvant treatment			0.450			0.098
No	75 (75%)	143 (70.1%)		131 (75.7%)	87 (66.4%)	
Yes	25 (25%)	61 (29.9%)		42 (24.3%)	44 (33.6%)	
Albumin (g/L)			0.917			0.709
Mean ± SD	40.762 ± 5.526	40.695 ± 5.164		40.816 ± 5.547	40.587 ± 4.915	
NPS			0.602			0.829
1	23 (23%)	44 (21.6%)		36 (20.8%)	31 (23.7%)	
2	47 (47%)	87 (42.6%)		78 (45.1%)	56 (42.7%)	
3	30 (30%)	73 (35.8%)		59 (34.1%)	44 (33.6%)	
PNI			0.731			0.131
Mean ± SD	50.872 ± 7.134	51.152 ± 5.606		50.597 ± 6.267	51.671 ± 5.937	
COUNT			0.621			0.477
Normal/light	75 (75%)	146 (71.6%)		129 (74.6%)	92 (70.2%)	
Moderate	25 (25%)	58 (28.4%)		44 (25.4%)	39 (29.8%)	

*Abbreviations*: *ALB* Serum albumin concentration, *BMI* Body mass index, *CONUT* Controlling nutritional status, *MAI* Muscle-adipose index, *PNI* Prognostic nutritional index, *SD* Standard deviation

### Analysis of the effect of MAI on the prognosis of ESCC patients

**Fig. 2:**
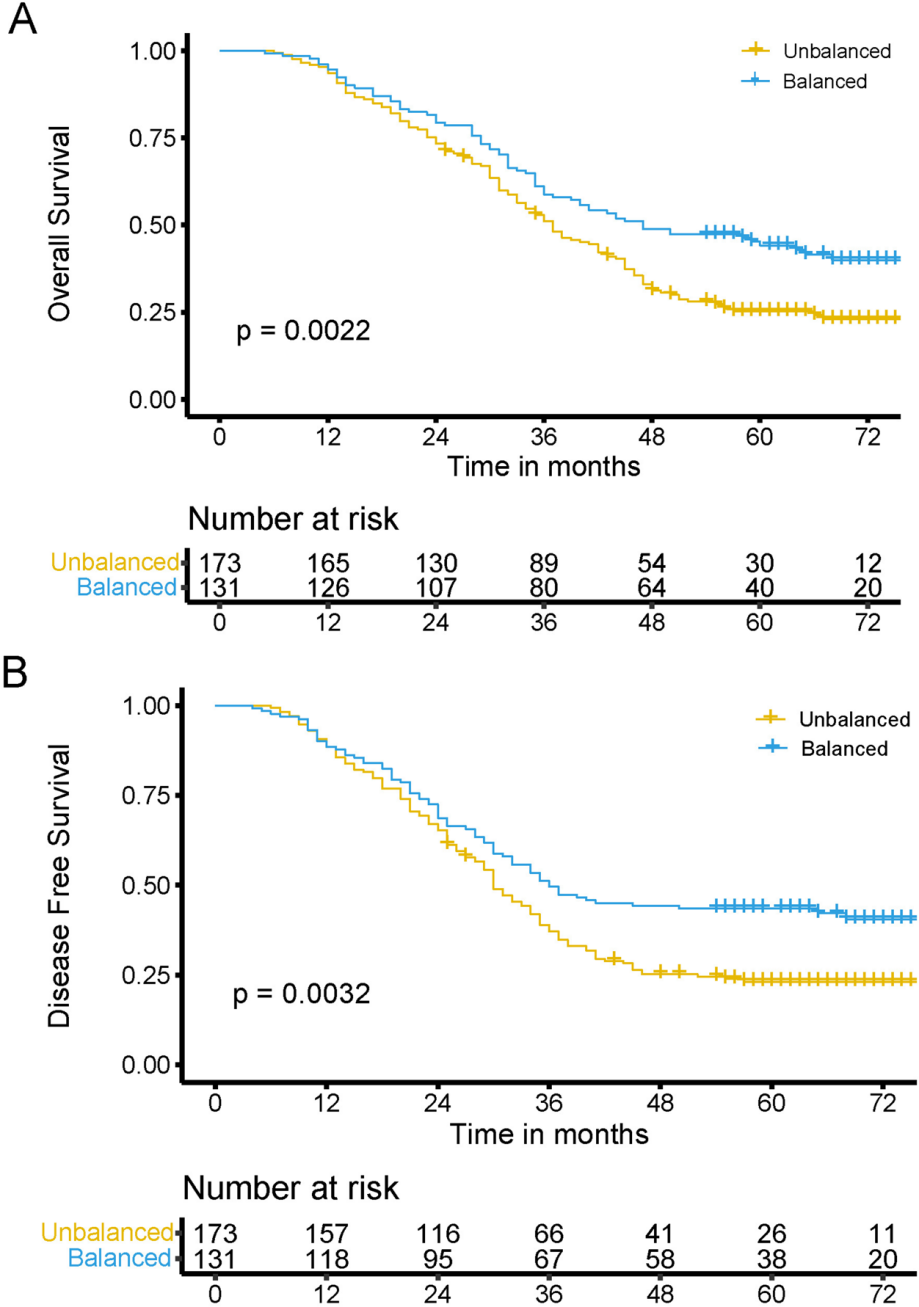
Kaplan–Meier analysis for overall survival (**A**) and disease-free survival (**B**) with or without muscle-adipose imbalance

X-tile analysis was performed to investigate the OS and DFS of patients with ESCC who underwent surgery. The aim of this study was to identify the minimum *P*-value of the log-rank test and establish a diagnostic cutoff value for the male- and female-specific muscle-adipose-imbalanced and balanced groups. The results indicated that patients in both female and male groups with a high MAI had better OS and DFS than those with a low MAI (all *P* < 0.05). In the female group, the diagnostic cutoff value was set at 0.6, and the survival (OS, *P* = 0.005; DFS, *P* = 0.028) of the balanced group (51 cases) was better than that of the imbalanced group (49 cases) (Supplementary Figs. 1, 2). In the male group, the diagnostic cutoff value was set at 2453.6, and the survival of the balanced group (117 cases) (OS, *P* = 0.035; DFS, *P* = 0.024) was better than that of the the imbalanced group (87 cases) (Supplementary Figs. 3, 4). Figure 2 displays the Kaplan–Meier curves of OS and DFS for ESCC patients in the imbalanced and balanced groups. The 5-year OS and DFS rates of the balanced group were significantly higher than those of the imbalanced group (OS: balanced, 44.2%; imbalanced, 25.3%, *P* = 0.002; DFS: balanced, 43.5%; imbalanced, 23.1%, *P* = 0.003).

**Fig. 3:**
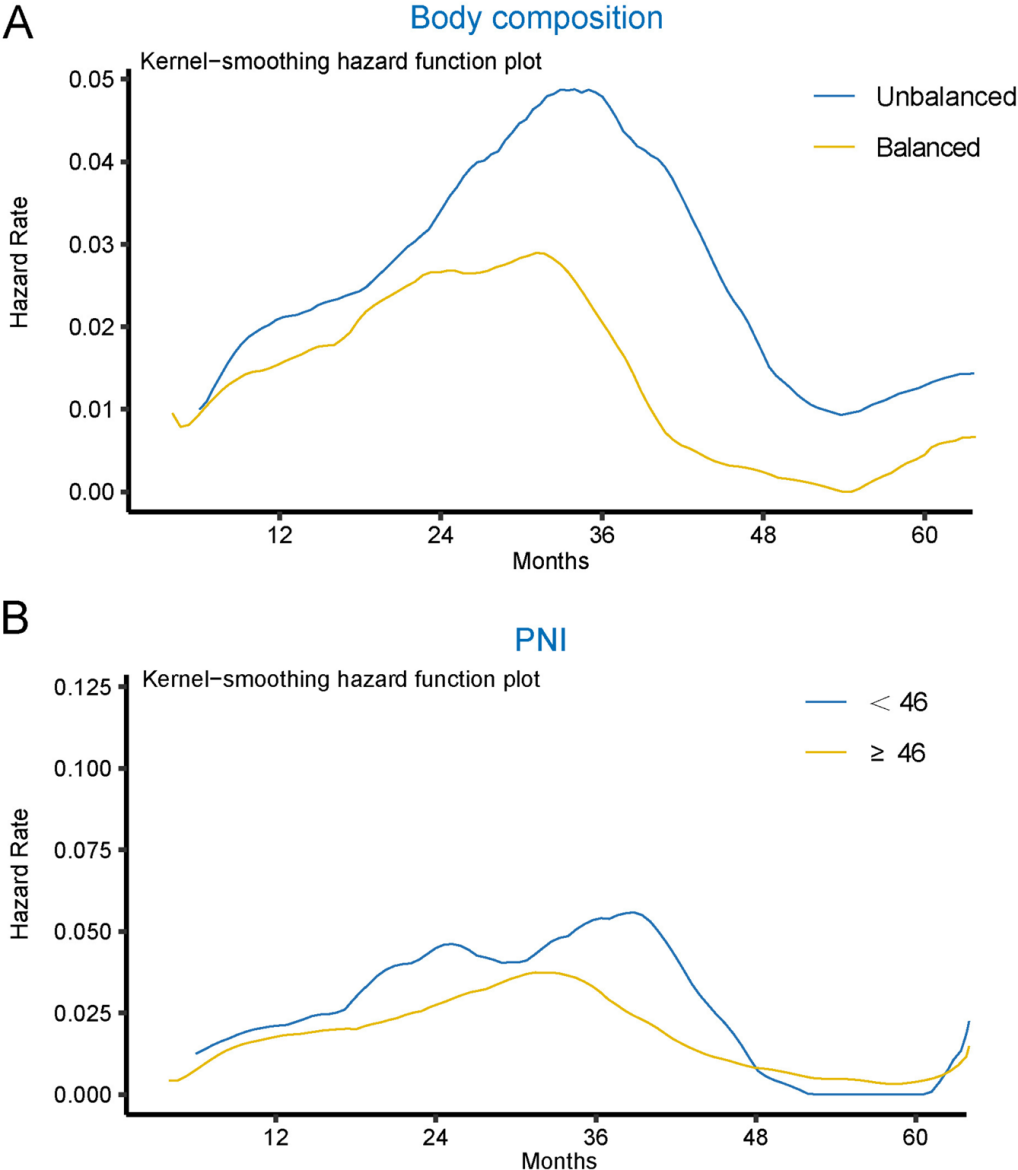
Hazard functions in all patients between the muscle-adipose imbalanced and balanced groups (**A**) and between the PNI < 46 group and the PNI ≥ 46 group (**B**)

**Fig. 4:**
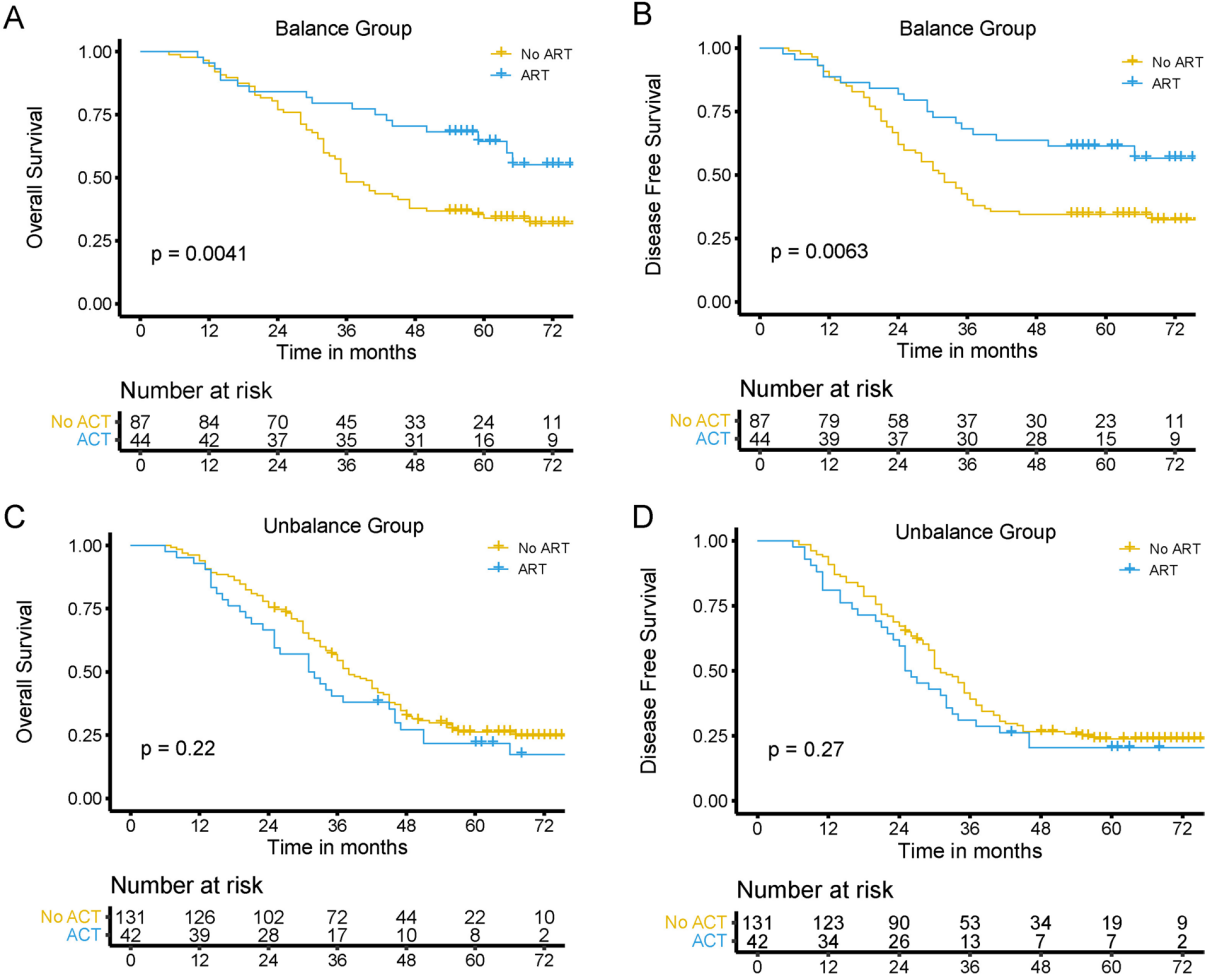
Kaplan–Meier analysis for overall survival (**A**) and disease free survival (**B**) in the muscle-adipose balanced group (**A**, **B**) and the muscle-adipose imbalanced group (**C**, **D**) according to with or without postoperative adjuvant radiotherapy (ART)

Univariate and multivariate analyses were performed using the Cox proportional hazards regression model to identify clinical characteristics that were significantly associated with OS (Table 2) and DFS (Table 3). The results indicated that tumor differentiation, TNM stage, PNI, NPS, and MAI served as independent prognostic markers for both overall survival (OS) and disease-free survival (DFS).

**Table 2:** Univariate and multivariate analysis for overall survival in all patients

Variable	Univariable		Multivariable	
	HR (95% CI)	*P*	HR (95% CI)	*P*
Gender				
Female	1			
Male	0.83 (0.62–1.10)	0.198		
Age (years)				
< 65	1			
≥ 65	0.90 (0.68–1.20)	0.484		
Tumor length (cm)				
< 3	1			
≥ 3	0.95 (0.70–1.30)	0.765		
Vessel invasion				
Negative	1			
Positive	1.06 (0.71–1.58)	0.769		
Perineural invasion				
Negative	1			
Positive	1.26 (0.90–1.76)	0.185		
Tumor location				
Upper	1			
Middle	1.02 (0.64–1.62)	0.939		
Lower	0.90 (0.55–1.47)	0.677		
Differentiation				
Well	1		1	
Moderate	1.48 (0.95–2.31)	0.081	1.39 (0.89–2.18)	0.151
Poor	2.32 (1.40–3.83)	0.001	1.94 (1.16–3.24)	0.012
TNM				
I	1		1	
II	1.28 (0.88–1.86)	0.203	1.21 (0.83–1.77)	0.322
III	1.73 (1.18–2.53)	0.005	1.51 (1.02–2.23)	0.039
Adjuvant treatment				
Yes	1			
No	1.32 (0.96–1.82)	0.089		
NPS				
0	1		1	
1	1.54 (1.03–2.29)	0.034	1.42 (0.95–2.12)	0.091
2	2.09 (1.39–3.15)	< 0.001	1.70 (1.11–2.59)	0.014
Albumin (g/L)				
< 35				
≥ 35	0.71 (0.50–1.01)	0.058		
PNI				
< 46	1		1	
≥ 46	0.63 (0.46–0.85)	0.003	0.71 (0.52–0.98)	0.036
COUNT				
Normal/light	1			
Moderate	1.22 (0.97–1.45)	0.064		
MAI				
Unbalance	1		1	
Balance	0.64 (0.48–0.85)	0.002	0.67 (0.50–0.90)	0.007

*Abbreviations*: *ALB* Serum albumin concentration, *BMI* Body mass index, *CI* Confidence interval, *CONUT* controlling nutritional status, *HR* Hazard ratio, *MAI* Muscle-adipose index, *PNI* Prognostic nutritional index, *SD* Standard deviation

**Table 3:** Univariate and multivariate analysis for disease-free survival in all patients

Variable	Univariable (DFS)		Multivariable (DFS)	
	HR (95% CI)	*P*	HR (95% CI)	*P*
Gender				
Female	1			
Male	0.84 (0.63–1.12)	0.242		
Age (years)				
< 65	1			
≥ 65	0.87 (0.66–1.15)	0.329		
Tumor length (cm)				
< 3	1			
≥ 3	0.97 (0.72–1.32)	0.867		
Vessel invasion				
Negative	1			
Positive	1.07 (0.72–1.59)	0.726		
Perineural invasion				
Negative	1			
Positive	1.24 (0.88–1.73)	0.214		
Tumor location				
Upper	1			
Middle	1.11 (0.69–1.76)	0.671		
Lower	0.97 (0.60–1.57)	0.889		
Differentiation				
Well	1		1	
Moderate	1.42 (0.92–2.19)	0.114	1.33 (0.86–2.07)	0.205
Poor	2.22 (1.36–3.64)	0.002	1.88 (1.14–3.11)	0.014
TNM				
I	1		1	
II	1.24 (0.86–1.79)	0.255	1.17 (0.80–1.70)	0.415
III	1.64 (1.13–2.38)	0.010	1.46 (1.00–2.15)	0.049
Adjuvant treatment				
Yes	1			
No	1.32 (0.96–1.81)	0.089		
NPS				
0	1		1	
1	1.40 (0.95–2.06)	0.088	1.30 (0.88–1.92)	0.194
2	1.90 (1.28–2.82)	0.001	1.59 (1.06–2.38)	0.025
Albumin (g/L)				
< 35				
≥ 35	0.79 (0.56–1.12)	0.179		
PNI				
< 46	1		1	
≥ 46	0.64 (0.47–0.87)	0.004	0.71 (0.52–0.96)	0.028
COUNT				
Normal/light	1			
Moderate	1.25 (0.86–1.55)	0.072		
MAI				
Unbalance	1		1	
Balance	0.65 (0.49–0.87)	0.003	0.70 (0.53–0.93)	0.014

*Abbreviations*: *ALB* Serum albumin concentration, *BMI* Body mass index, *CI* Confidence interval, *CONUT* Controlling nutritional status, *HR* Hazard ratio, *MAI* Muscle-adipose index, *PNI* Prognostic nutritional index, *SD* Standard deviation

### Influence of the MAI and PNI on immediate postoperative recurrence risk

The influence of MAI and PNI on immediate postoperative recurrence risk in ESCC cases was further analyzed. The findings depicted in Fig. 3A demonstrate that the risk of postoperative recurrence in the muscle-adipose-imbalance-group was significantly greater than that in the balanced group (imbalanced: 0.048, balanced: 0.029). Patients in the imbalanced group showed a higher propensity for recurrence than those in the balanced group. Instantaneous recurrence risk after surgery was assessed using the PNI. The nuclear density curve in Fig. 3B reveals that owing to their mixed characteristics, the recurrence risk in both groups could not be stratified after approximately 4 years.

### Impact of the MAI on the benefits of postoperative radiotherapy

The association between the MAI and the benefits of postoperative RT was further explored. The results showed that muscle-adipose-balanced ESCC patients with risk factors could benefit from postoperative RT in both OS (*P* = 0.004) and DFS (*P* = 0.006) (Fig. 4 A, B). No significant survival benefit from adjuvant RT was found in the muscle-adipose-imbalanced group (OS, *P* = 0.22; DFS, *P* = 0.27) (Fig. 4 C, D).

### Nomogram construction and model comparison

**Fig. 5:**
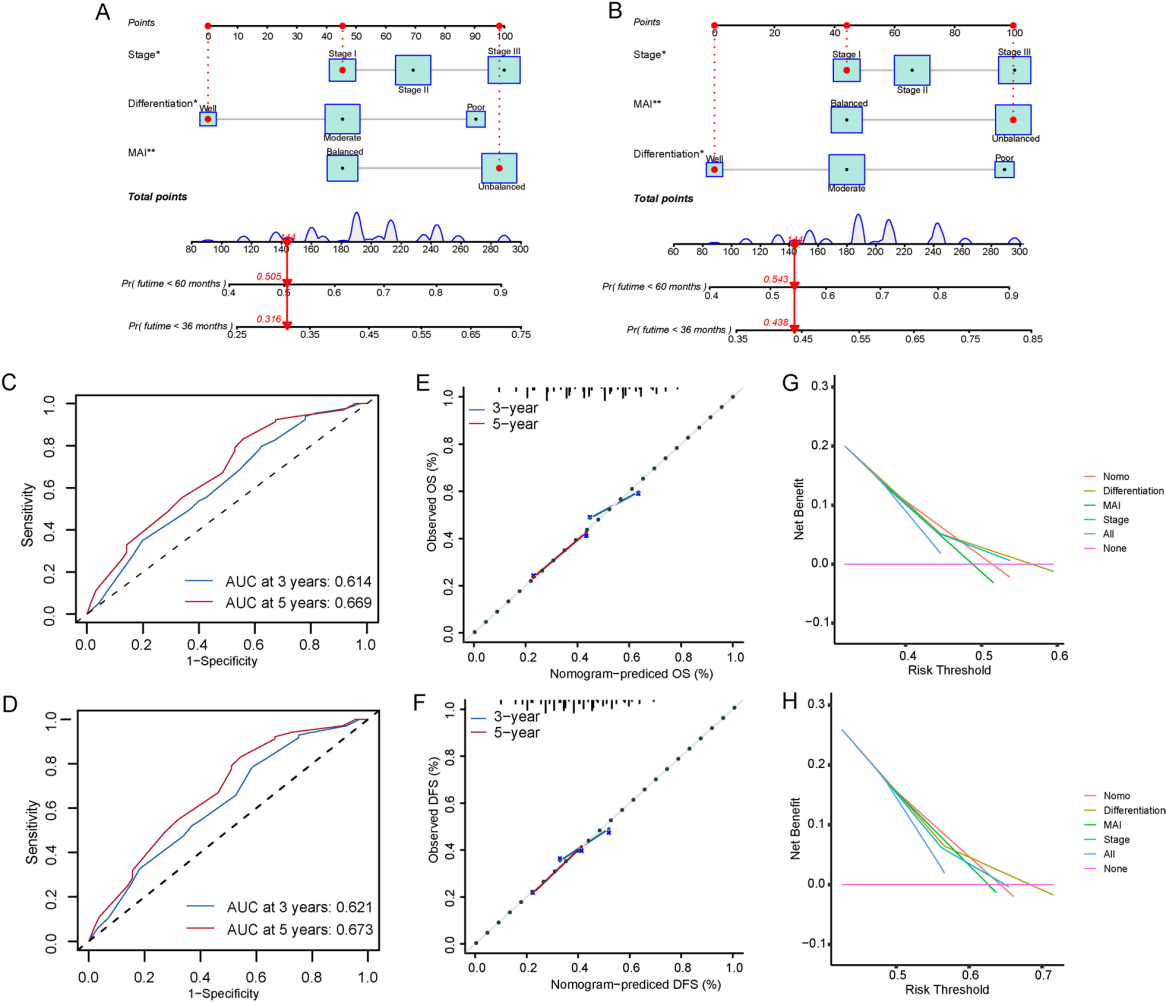
Nomogram predicting the overall survival (**A**) and disease-free survival (**B**) of patients with resected esophageal squamous cell carcinoma. Area under the receiver operating characteristic curve for 3- and 5-year overall survival (**C**) and disease-free survival (**D**) Calibration curves show good consistency in the probabilities of 3- and 5-year overall survival (**E**) and disease-free survival (**F**) between the nomogram predictions and the actual observations. Decision curve analysis comparing the OS (**G**) and DFS (**H**) of the nomogram with tumor differentiation, TNM stage, and MAI alone, based on the threshold probability

The results obtained through multivariate analyses, tumor differentiation, TNM stage, and MAI were used to establish a nomogram to predict 3- and 5-year OS and DFS for patients with ESCC (Fig. 5A, B). The C-indexes of the nomograms for OS and DFS were 0.67 and 0.68, respectively. The areas under the curves (AUCs) for 3- and 5-year OS were 0.614 and 0.669, respectively (Fig. 5C). Figure 5D shows that the AUCs for the 3- and 5-year DFS were 0.621 and 0.673, respectively. Good consistency concerning the 3- and 5-year OS and DFS probabilities was observed in the calibration curves between the actual observations and nomogram predictions (Fig. 5E, F). Decision curve analysis was used to compare the clinical efficacy (OS and DFS) of the nomogram with that of tumor differentiation, TNM stage, and MAI alone based on the threshold probability. The nomogram provided an excellent predictive model superior to that of tumor differentiation, TNM stage, and MAI alone (Fig. 5G, H), which was consistent with the results of the receiver operating curve (ROC) curve comparisons (Supplementary Figs. 5, 6).

## Discussion

In this study, we introduced an innovative male/female-specific BCI, referred to as the MAI, which effectively combines muscle and adipose components. The association between this BCI and survival prognosis in ESCC was analyzed and found found that MAI could serve as an independent prognostic factor of OS and DFS. Furthermore, a novel nomogram incorporating MAI and other independent prognostic factors was conducted. This study highlights the importance of conducting body-composition assessment prior to surgery to provide individualized survival prediction and treatment strategies for patients with resected ESCC.

Previous studies have confirmed that body composition is closely associated with the survival of patients with several malignancies. Bekki et al. found that hepatocellular carcinoma patients with a low skeletal muscle index (SMI) prior to surgery had poor OS [21]. Preoperative SMI can serve as an independent disease recurrence indicator in colon cancer [22]. Oguma et al. observed that sarcopenia is an independent prognostic indicator of OS and DFS in patients [23]. However, another study by Kemper et al. showed a negative relationship between SMI before treatment and OS in patients with EC [24]. Two meta-analysis revealed that pretreatment sarcopenia was significantly associated with poor OS in EC patients [25, 26]. In addition, high levels of visceral adipose tissue were positively associated with the risk of recurrence in patients with colorectal cancer [27, 28]. In contrast, a negative relationship was observed between visceral adipose tissue levels and postoperative prognosis in patients with upper gastrointestinal cancer [29]. Matsui et al. conducted a meta-analysis to explore the association between adipose tissue levels and postoperative prognosis in patients with gastrointestinal cancer [30]. Results showed that high visceral fat levels were closely associated with improved OS after surgery in patients with gastrointestinal cancer. However, many patients with digestive tumors suffer from eating disorders and malnutrition, leading to the simultaneous loss of adipose and muscle tissues, especially in EC. Hagens et al. found that the levels of adipose and muscle tissue were closely associated with survival prognosis, and the combination of these two indicators had better prognostic accuracy than any single indicator [31, 32]. Therefore, in this study of patients with resected ESCC, we introduced the MAI that integrates two BCIs, muscle and adipose. This novel index is sex specific and aims to provide clinicians with a comprehensive tool for individual survival prediction and treatment-strategy development.

Traditional nutritional indicators based on blood chemistry and cells are crucial parameters that provide valuable insights into the nutritional status of patients prior to surgery. The CONUT score, calculated using ALB, plasma cholesterol, and total lymphocyte count in the blood, was confirmed as a survival prognosis indicator in EC patients undergoing esophagectomy [33]. The results of another meta-analysis revealed that PNI could be utilized for survival prediction in patients with EC, and a lower PNI was an unfavorable prognostic factor closely associated with poor prognosis [34]. A retrospective study by Feng et al. demonstrated that the NPS could serve as a valuable prognostic indicator for individual survival prediction in resected ESCC patients [18]. The CONUT score, PNI, and NPS were also analyzed in our data cohort, and the prognostic prediction trends of these three nutritional indicators were consistent with those of previous studies. However, other factors, including blood volume, autoimmune and inflammatory conditions, etc., also influence these nutritional indicators [35, 36]. The MAI might be considered more objective than unstable nutritional indicators based on hematological factors. In this study, the MAI and PNI were identified as independent prognostic indicators after univariate and multivariate analyses.

Although the Kaplan–Meier method is an essential tool for demonstrating cumulative probabilities over time, the hazard function provides valuable information on the instantaneous risk of recurrence. The MAI outperformed the PNI in assessing the risk of recurrence, as shown in our further analyses. The instantaneous recurrence risk of patients with ESCC could be well stratified by the MAI, whereas the PNI was confounded after a 4-year follow-up period. Our findings indicate that utilizing the MAI for preoperative nutritional assessment may be a superior alternative to using the PNI. By incorporating multiple factors and assessing their individual weights, the nomogram can enhance the prediction accuracy and practicability. In this study, three variables—tumor differentiation, TNM stage, and MAI—were selected to establish the nomogram, facilitating its application in clinical practice.

Recently, the significant influence of body composition on the prognosis of patients undergoing RT has attracted increasing attention. For example, Iwashita et al. found that sarcopenia is closely associated with poor survival in patients with EC receiving radical irradiation [37]. Another retrospective study demonstrated that sarcopenia is an effective indicator of prognosis in patients with ESCC undergoing RT [38]. Poor tolerance and frequent RT breaks in patients with sarcopenia may result in poor prognosis. In our study, we further analyzed the influence of the MAI on the benefits of adjuvant RT. We found that patients with muscle-adipose-balanced ESCC benefited from postoperative RT.

In this study, the survival prognostic value of the MAI was explored for the first time (to our knowledge) in resected ESCC, and a nomogram based on the MAI and other clinical characteristics was constructed and demonstrated exceptional accuracy and effectiveness as a tool for survival prediction. However, this study had several limitations. First, a potential bias could not be avoided due to its retrospective nature. Second, the application of adjuvant RT in our study was non-randomized, and information on the proportion of patients receiving concomitant RT was unknown, leading to possible selection bias. Third, the causal associations could not be defined in any observational study.

## Conclusion

In summary, the current study aimed to establish an association between the MAI and survival outcomes (OS and DFS) in patients with resected ESCC. The MAI has significant value in predicting postoperative instantaneous recurrence risk and contributes to the construction of a new prognostic predictive model for patients with ESCC undergoing esophagectomy. Additionally, muscle-adipose-balanced ESCC may benefit from postoperative RT. However, future validation using large-scale external data is warranted to confirm the conclusions of this study.

### Supplementary Information

**Supplementary Material 1.**

## Data Availability

No datasets were generated or analysed during the current study.

## Abbreviations

CT: Computed tomography
MAI: Muscle adipose index
OS: Overall survival
DFS: Disease-free survival
PNI: Prognostic nutritional index
ESCC: Esophageal squamous cell carcinoma
EC: Esophageal carcinoma
BMI: Body mass index
CONUT: Controlling nutritional status
ALB: Albumin
BCI: Body composition index
NPS: Naples prognostic score
TC: Total cholesterol
HR: Hazard rate
ROC: Receiver operating characteristic
AUC: Area under the curve
SMI: Skeletal muscle index
RT: Radiotherapy

## References

[1] GBD 2019 Adolescent Young Adult Cancer Collaborators (2022). The global burden of adolescent and young adult cancer in 2019: a systematic analysis for the global burden of disease study 2019. Lancet Oncol..

[2] Morgan E, Soerjomataram I, Rumgay H, Coleman HG, Thrift AP, Vignat J (2022). The global landscape of esophageal squamous cell carcinoma and esophageal adenocarcinoma incidence and mortality in 2020 and projections to 2040: new estimates from GLOBOCAN 2020. Gastroenterology.

[3] Rogers JE, Sewastjanow-Silva M, Waters RE, Ajani JA (2022). Esophageal cancer: emerging therapeutics. Expert Opin Ther Targets.

[4] Wang PY, Chen XK, Liu Q, Xu L, Zhang RX, Liu XB, Li Y (2021). Application of four nutritional risk indexes in perioperative management for esophageal cancer patients. J Cancer Res Clin Oncol.

[5] Deftereos I, Kiss N, Isenring E, Carter VM, Yeung JM (2020). A systematic review of the effect of preoperative nutrition support on nutritional status and treatment outcomes in upper gastrointestinal cancer resection. Eur J Surg Oncol.

[6] Caan BJ, Cespedes Feliciano EM, Prado CM, Alexeeff S, Kroenke CH, Bradshaw P (2018). Association of Muscle and Adiposity Measured by computed tomography with survival in patients with nonmetastatic breast cancer. JAMA Oncol.

[7] Lu J, Xue Z, Xie JG, Xu BB, Yang HB, Wu D (2022). Preoperative muscle-adipose index: a new prognostic factor for gastric cancer. Ann Surg Oncol.

[8] Holmes CJ, Racette SB (2021). The utility of body composition assessment in nutrition and clinical practice: an overview of current methodology. Nutrients.

[9] Onodera T, Goseki N, Kosaki G (1984). Prognostic nutritional index in gastrointestinal surgery of malnourished cancer patients. Nihon Geka Gakkai Zasshi.

[10] Jiang N, Deng JY, Ding XW, Ke B, Liu N, Zhang RP, Liang H (2014). Prognostic nutritional index predicts postoperative complications and long-term outcomes of gastric cancer. World J Gastroenterol.

[11] Kuroda D, Sawayama H, Kurashige J, Iwatsuki M, Eto T, Tokunaga R (2018). Controlling Nutritional status (CONUT) score is a prognostic marker for gastric cancer patients after curative resection. Gastric Cancer.

[12] Galizia G, Lieto E, Auricchio A, Cardella F, Mabilia A, Podzemny V (2017). Naples prognostic score, based on nutritional and inflammatory status, is an independent predictor of long-term outcome in patients undergoing surgery for colorectal cancer. Dis Colon Rectum.

[13] Zheng ZF, Lu J, Zheng CH, Li P, Xie JW, Wang JB (2017). A novel prognostic scoring system based on preoperative sarcopenia predicts the long-term outcome for patients after R0 resection for gastric cancer: experiences of a high-volume center. Ann Surg Oncol.

[14] Kim JH, Chin HM, Hwang SS, Jun KH (2014). Impact of intra-abdominal fat on surgical outcome and overall survival of patients with gastric cancer. Int J Surg.

[15] Li S, Qiu R, Yuan G, Wang Q, Li Z, Li Q, Zhang N (2022). Body composition in relation to postoperative anastomotic leakage and overall survival in patients with esophageal cancer. Nutrition.

[16] SliceOmatic [computer program]. Version 5.0. Montreal: TomoVision; 2015.

[17] Okugawa Y, Toiyama Y, Yamamoto A, Shigemori T, Ide S, Kitajima T (2020). Lymphocyte-C-reactive protein ratio as promising new marker for predicting surgical and oncological outcomes in colorectal cancer. Ann Surg.

[18] Feng JF, Zhao JM, Chen S, Chen QX (2021). Naples prognostic score: a novel prognostic score in predicting cancer-specific survival in patients with resected esophageal squamous cell carcinoma. Front Oncol.

[19] Yang YS, Shang QX, Yuan Y, Wu XY, Hu WP, Chen LQ (2019). Comparison of long-term quality of life in patients with esophageal cancer after Ivor-Lewis, McKeown, or sweet esophagectomy. J Gastrointest Surg.

[20] Helminen O, Mrena J, Sihvo E (2019). Can we increase the resection rate by minimally invasive approach? experience from 100 minimally invasive esophagectomies. J Oncol.

[21] Bekki T, Abe T, Amano H, Hattori M, Kobayashi T (2020). Impact of low skeletal muscle mass index and perioperative blood transfusion on the prognosis for HCC following curative resection. BMC Gastroenterol.

[22] Schaffler-Schaden D, Mittermair C, Birsak T, Weiss M, Hell T, Schaffler G, Weiss H (2020). Skeletal muscle index is an independent predictor of early recurrence in non-obese colon cancer patients. Langenbecks Arch Surg.

[23] Oguma J, Ozawa S, Kazuno A, Yamamoto M, Ninomiya Y, Yatabe K (2019). Prognostic significance of sarcopenia in patients undergoing esophagectomy for superficial esophageal squamous cell carcinoma. Dis Esophagus..

[24] Kemper M, Molwitz I, Krause L, Reeh M, Burdelski C, Kluge S (2021). Are muscle parameters obtained by computed tomography associated with outcome after esophagectomy for cancer?. Clin Nutr.

[25] Yao L, Wang L, Yin Y, Che G, Yang M (2022). Prognostic value of pretreatment skeletal muscle mass index in esophageal cancer patients: a meta-analysis. Nutr Cancer.

[26] Wang P, Wang S, Li X, Lin G, Ma Y, Xiao R (2022). Skeletal muscle wasting during neoadjuvant therapy as a prognosticator in patients with esophageal and esophagogastric junction cancer: a systematic review and meta-analysis. Int J Surg.

[27] Guiu B, Petit JM, Bonnetain F, Ladoire S, Guiu S, Cercueil JP (2010). Visceral fat area is an independent predictive biomarker of outcome after first-line bevacizumab-based treatment in metastatic colorectal cancer. Gut.

[28] Moon HG, Ju YT, Jeong CY, Jung EJ, Lee YJ, Hong SC (2008). Visceral obesity may affect oncologic outcome in patients with colorectal cancer. Ann Surg Oncol.

[29] Harada K, Baba Y, Ishimoto T, Kosumi K, Tokunaga R, Izumi D (2015). Low visceral fat content is associated with poor prognosis in a database of 507 upper gastrointestinal cancers. Ann Surg Oncol.

[30] Matsui R, Watanabe J, Banno M, Inaki N, Fukunaga T (2022). Association of visceral adipose tissue with postoperative outcome in upper gastrointestinal cancer: a systematic review and meta-analysis. Am J Clin Nutr.

[31] Hagens ERC, Feenstra ML, van Egmond MA, van Laarhoven HWM, Hulshof MCCM, Boshier PR (2020). Influence of body composition and muscle strength on outcomes after multimodal oesophageal cancer treatment. J Cachexia Sarcopenia Muscle.

[32] Caan BJ, Meyerhardt JA, Kroenke CH, Alexeeff S, Xiao J, Weltzien E (2017). Explaining the obesity paradox: the association between body composition and colorectal cancer survival (C-SCANS study). Cancer Epidemiol Biomarkers Prev.

[33] Takagi K, Buettner S, Ijzermans JNM, Wijnhoven BPL (2020). Systematic review on the Controlling Nutritional status (CONUT) score in patients undergoing esophagectomy for esophageal cancer. Anticancer Res..

[34] Liao G, Zhao Z, Yang H, Chen M, Li X (2020). Can prognostic nutritional index be a prediction factor in esophageal cancer?: a meta-analysis. Nutr Cancer.

[35] de Ulíbarri Pérez JI, Fernández G, Rodríguez Salvanés F, Díaz López AM (2014). Nutritional screening; control of clinical undernutrition with analytical parameters. Nutr Hosp.

[36] Cengiz O, Kocer B, Sürmeli S, Santicky MJ, Soran A (2006). Are pretreatment serum albumin and cholesterol levels prognostic tools in patients with colorectal carcinoma?. Med Sci Monit..

[37] Iwashita K, Kubota H, Nishioka R, Emoto Y, Kawahara D, Ishihara T (2023). Prognostic value of radiomics analysis of skeletal muscle after radical irradiation of esophageal cancer. Anticancer Res..

[38] Qian J, Si Y, Zhou K, Tian Y, Guo Q, Zhao K, Yu J (2022). Sarcopenia is associated with prognosis in patients with esophageal squamous cell cancer after radiotherapy or chemoradiotherapy. BMC Gastroenterol.

