# Supplementary material for: Association of preoperative muscle-adipose index measured by computed tomography with survival in patients with esophageal squamous cell carcinoma
Source: World J Surg Oncol. 2024 Feb 22;22:61. doi: 10.1186/s12957-024-03338-0 (PMC10882774; doi:10.1186/s12957-024-03338-0)

**Association of Preoperative Muscle-Adipose Index Measured by Computed Tomography with Survival in Patients With Esophageal Squamous Cell Carcinoma**

Danqi Qian, Peipei Shen, Jiahao Zhu, Shengjun Ji, Yan Kong

**Supplementary Figures**

Supplementary Figure 1. X-tile plots: The effect of the muscle-adipose index on the Kaplan-Meier analysis of postoperative OS in female patients (*P* = 0.005).


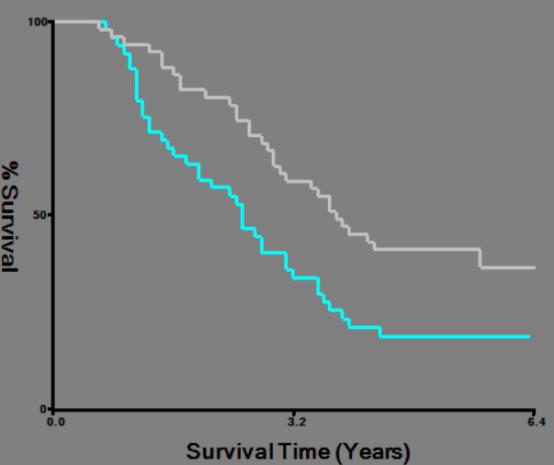


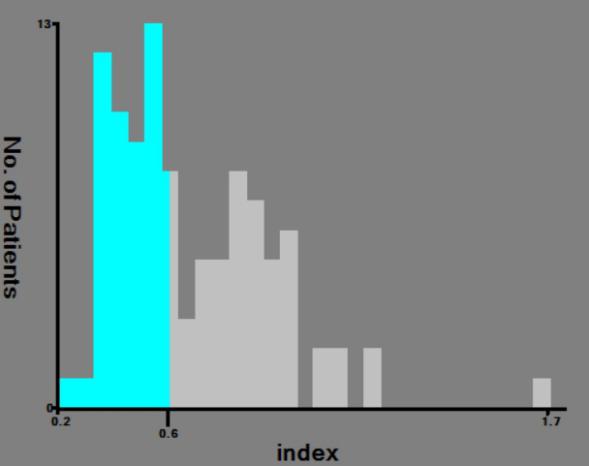


Supplementary Figure 2. X-tile plots: The effect of the muscle-adipose index on the Kaplan-Meier analysis of postoperative DFS in female patients (*P* = 0.028).


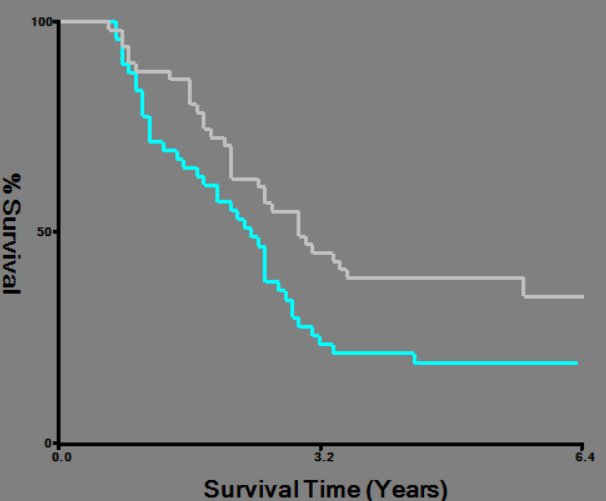

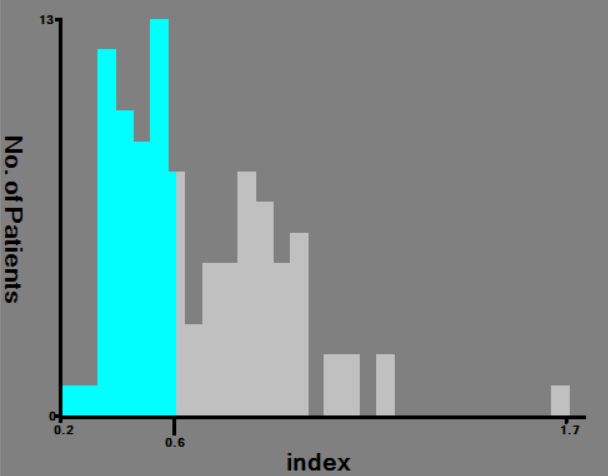


Supplementary Figure 3. X-tile plots: The effect of the muscle-adipose index on the Kaplan-Meier analysis of postoperative OS in male patients (*P* = 0.035).


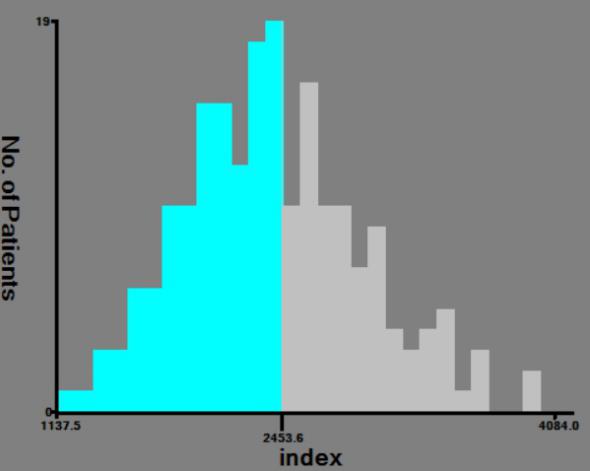

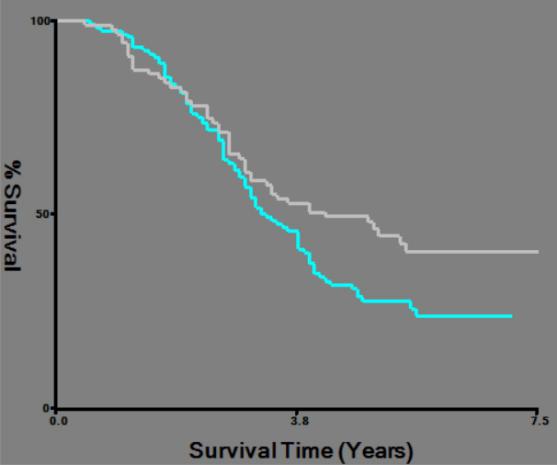


Supplementary Figure 4. X-tile plots: The effect of the muscle-adipose index on the Kaplan-Meier analysis of postoperative DFS in male patients (*P* = 0.024).


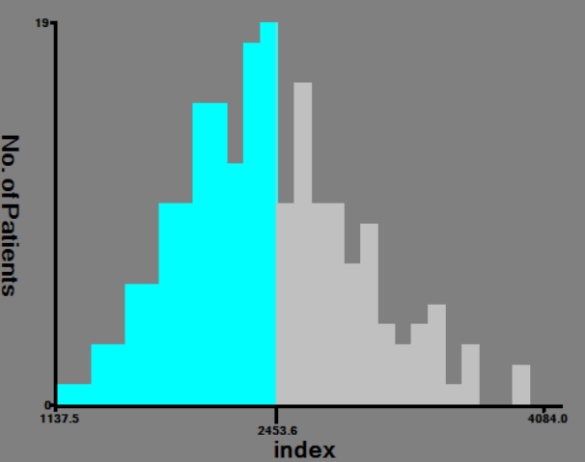

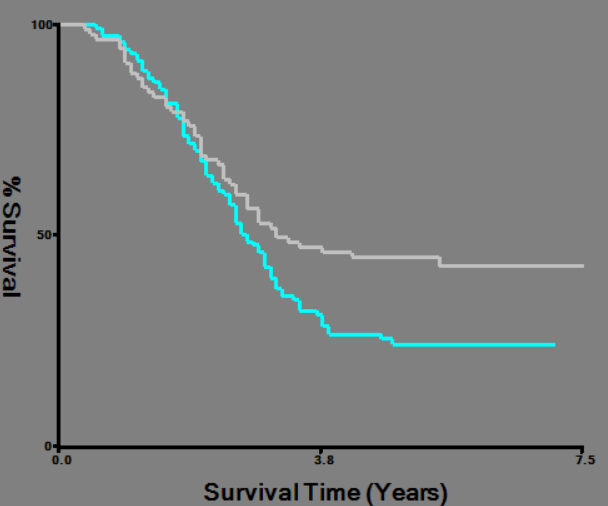


Supplementary Figure 5. ROC analyses and AUC comparison among different models predicting tumor mortality.


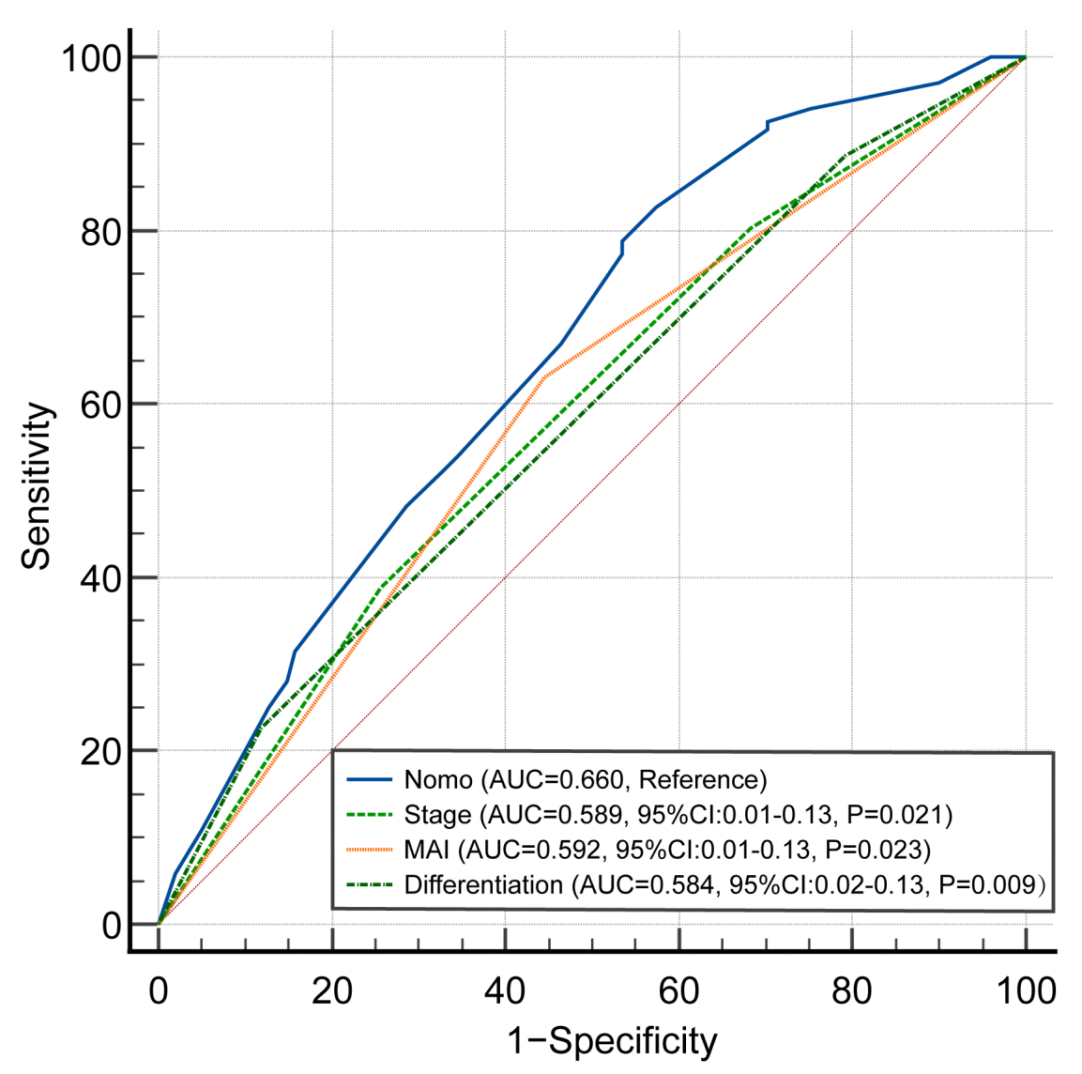


Supplementary Figure 6. ROC analyses and AUC comparison among different models predicting tumor recurrence.


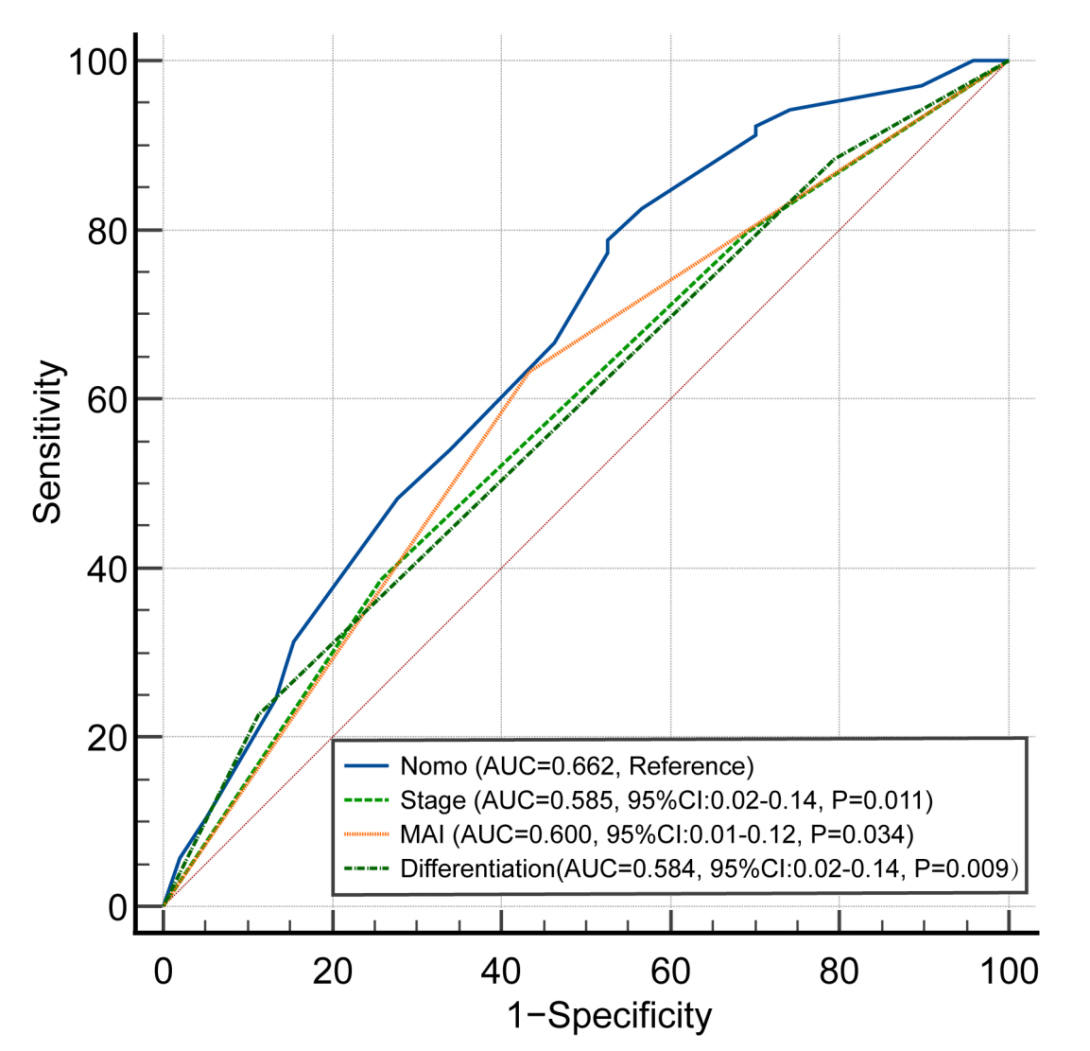

Supplement: Supplementary file 1 — Supplementary Material 1. [file 12957_2024_3338_MOESM1_ESM.docx]
